# Supplementary material for: Phytoplankton and Bacterial Community Structure in Two Chinese Lakes of Different Trophic Status
Source: Microorganisms. 2019 Nov 27;7(12):621. doi: 10.3390/microorganisms7120621 (PMC6956004; doi:10.3390/microorganisms7120621)
Supplement: Supplementary file 1 [file microorganisms-07-00621-s001.pdf]

Supplemental Marterials

Table S1 The community composition of phytoplankton in oligotrophic Lake Basomtso and eutrophic Lake South.

Phylum	Genus /Species	Oligotrophic (Lake Basomtso)	Eutrophic (Lake South)
Cyanobacteria	*Dolichospermum circinale*	+	+ + +
	Dolichospermum viguieri		+ + +
	Pseudanabaena sp.		+ + +
	Arthrospira platensis		+
	Anabaenopsis arnoldii Aptek		+ +
	Microcystis flosaquae		+
	Microcystis wesenbergii		+ + +
	Chroococcus sp.		+ +
	Chroococcus turgidus	+	
	Chroococcus minutus		+
	Chroococcus minor		+
	Oscillatoria sp.		+
	Oscillatoria limosa	+	
	Oscillatoria okenii	+	
	Merismopedia tranquilla		+ +
	Merismopedia minima		+ ++
	Merismopedia glauca		+
	Coelosphaerium dubium		+
	Dactylococcopsis sp.		+
	Aphanothece sp.		+
	Aphanocapsa sp.		+ +
Pyrrophyta	*Nusuttodinium aeruginosum*		+
	Peridinium sp.		+
	Peridiniopsis elpatiewskyi	+	
Cryptophyta	*Cryptomonas ovata*		+
	Cryptomonas erosa		+
	Chroomonas sp.		+
Chrysophyta	*Dinobryon divergens*	+	
Bacillariophyta	*Cyclotella meneghiniana*	+ ++	+ ++
	Discostella stelligera		+ +
	Aulacoseira granulata	+	+
	Aulacoseira granulata var. angustissima		+ +
	Asterionella sp.		+ ++
	Ctenophora pulchella	+	+
	Nitzschia holsatica		+
	Ulnaria ulna		+
	Ulnaria danica	+	
	Ulnaria acus	+	+
	Ulnaria capitata	+	
	Navicula sp.	+ + +	+ +
	Fragilaria crotonensis	+	
	Fragilaria crotonensis var.oregona	+	

Phylum	Genus /Species	Oligotrophic (Lake Basomtso)	Eutrophic (Lake South)
	Hannaea arcus	+	
	Diatoma vulgaris var. vulgaris	+	
	Eunoria sp.	+	
	Gyrosigma sp.	+	
	Diploneis elliptica	+	
	Cymbella tumida	+	
	Nitzschia linearis	+ + +	
	Gomphonema parvulum	+	
	Gomphonema truncatum var. turgidum	+	
	Iconella biseriata	+ + +	
Euglenophyta	*Lepocinclis acus*		+
	Khawkinea acutecaudata		+
	Euglena gasterosteus		+
	Euglena viridis		+
	Euglena deses		+
	Monomorphina pyrum		+
	Phacus pleuronectes		+
	Phacus longicauda		+
	Phacus triqueter		+
	Trachelomonas sp.	+	
	Trachelomonas oblanga		+
	Strombomonas rotunda		+
	Strombomonas fluviatilis		+
Chlrophyta	*Monactinus simplex*		+ +
	Pediastrum duplex		+
	Lacunastrum gracillimum		+
	Stauridium tetras		+ +
	Parapediastrum biradiatum		+
	Actinastrum sp.		+
	Chlorella sp.		+
	Tetraedron trigonum		+
	Tetraedron trigonum var.gracile		+
	Tetraedron trilobulatum		+
	Tetraedron caudatum		+
	Pseudostaurastrum hastatum		+
	Tetraedron minimum		+
	Pandorina morum		+
	Eudorina elegans		+
	Staurodesmus sp.		+
	Gonatozygon sp.	+	
	Cosmarium laeve		+
	Scenedesmus sp.		+
	Scenedesmus quadricauda		+
	Desmodesmus abundans		+ +
	Tetradesmus dimorphus		+
	Scenedesmus denticulatus		+
	Scenedesmus bicaudatus		+ +

Phylum	Genus /Species	Oligotrophic (Lake Basomtso)	Eutrophic (Lake South)
	Tetradesmus obliquus		+
	Coelastrum microporum		+
	Coelastrum sphaericum		+
	Kirchneriella lunaris		+
	Selenastrum bibraianum		+
	Monoraphidium minutum		+
	Tetrallantos lagerheimii		+
	Closterium gracile		+
	Crucigenia quadrata		+ +
	Willea rectangularis		+ +
	Lemmermannia komarekii		+
	Crucigenia tetrapedia		+
	Oocystis lacustris		+ +
	Neglectella solitaria		+
	Pteromonas aculeata var. aculeata		+
	Pteromonas golenkiniana var. subquadrata		+
	Chlamydomonas sp.		+
	Chlamydomonas proboscigera		+
	Ankistrodesmus falcatus		+
	Monoraphidium griffithii		+
	Tetrastrum staurogeniaeforme		+
	Pseudoschroederia robusta		+
	Schroederia spiralis		+
	Schroederia nitzschioides		+
	Westella sp.		+
	Lagerheimia wratislawiensis		+
	Lagerheimia subsalsa Lemmermann		+
	Dictyosphaerium sp.		+
	Treubaria crassispina		+
**Total number**		**27**	**97**

Notes: +++ represents dominant species; ++ represents common species; + general species
